# Where Cost of Food Hits Hardest: Investigation of Diet Cost and Affordability in a Low Socioeconomic Region of Australia

**DOI:** 10.1002/hpja.70092

**Published:** 2025-09-02

**Authors:** Samantha Dean, Meron Lewis, Karen Walton, Katherine Kent, Karen E. Charlton

**Affiliations:** ^1^ Faculty of Science, Medicine and Health, School of Medical, Indigenous and Health Sciences University of Wollongong Wollongong Australia; ^2^ Faculty of Medicine, School of Human Movement and Nutrition Science University of Queensland St Lucia Queensland Australia

**Keywords:** affordability, community survey, cost analysis, food policy, food security, low‐income population, socioeconomic factors

## Abstract

**Introduction:**

Residents in the Fowler electorate of NSW, Australia experience high socioeconomic disadvantage and may therefore be vulnerable to food insecurity. This study aimed to assess the cost, cost differential and affordability of recommended and current diets for various household structures in this electorate.

**Methods:**

This study applied the low socioeconomic group Healthy Diets Australian Standardised Affordability and Pricing protocol. Food and beverage prices, including both ‘popular brand’ and ‘cheapest alternative’ options, were collected from 43 outlets in five locations across Fowler using standardised recommended and current diet pricing tools. Fortnightly diet costs and the differential between both diets and pricing options were calculated for a family of four, a single‐parent family and a single male. Diet affordability was assessed against low‐minimum wage and welfare‐dependent household incomes, characterising diet costs as causing ‘food stress’ or being ‘unaffordable’ if exceeding 25% and 30% of household income, respectively.

**Results:**

Recommended diets were less expensive than current diets for all households by 9%–31%. Pricing ‘cheapest alternatives’ reduced both diet costs by 30%–34%.

For ‘popular brands’, recommended and current diets required 13%–34% and 19%–42% of household income, respectively, while ‘cheapest alternatives’ required 9%–23% and 13%–28% of household income, respectively. Recommended and current diets priced with ‘popular brands’ were unaffordable or caused ‘food stress’ for many welfare‐dependent and low‐income families with children.

**Conclusion:**

Whilst recommended diets were less expensive than current diets, they were unaffordable or caused ‘food stress’ for many welfare‐dependent and low‐income families with children unless the households purchased the ‘cheapest alternatives’.

**Implications for Health Promotion:**

Targeted policy interventions to improve diet affordability for regions with high socioeconomic disadvantage are urgently required, including expansion of local‐level food access initiatives and, more broadly, stronger fiscal policy measures to address dietary inequities.

## 1 Introduction

Food security is recognised as a fundamental human right [1]. Food security is defined as, ‘when all people at all times have physical and economic access to sufficient, safe and nutritious food to meet their dietary needs and food preferences for an active and healthy life’ [2]. Underpinning this definition are six fundamental pillars—availability, access, utilisation, stability, sustainability and agency [3]. The disruption or absence of any of these pillars results in *food insecurity* [4]. Depending on the level of severity, food insecurity can range from anxiety that food will run out (marginal) to a decrease in the quality, quantity and variety of selected foods (moderate) to disruptions to an individual's eating patterns (severe) [5]. Food insecurity has been linked to an increased risk of chronic conditions, reduced well‐being and in severe cases, nutritional deficiencies and delayed development in children [6]. Globally, food insecurity is a persistent challenge, with increasing rates in high‐income countries, likely driven by inflationary pressures from events including the Ukraine war, the COVID‐19 pandemic and supply chain disruptions resulting from extreme weather events [7].

Within Australia, the rising cost of living is a significant contributor to the growing issue of food insecurity [5]. During 2023–2024, an estimated 3.4 million Australian households reported moderate to severe food insecurity [5]. Food insecurity amongst low‐income households rose to almost half (48%), representing a 5% rise in the proportion of affected households since 2022 [5]. Within Australia, certain demographic groups face elevated risks of food insecurity, including those who are unemployed, single‐parent households, those renting accommodation and individuals from diverse cultural backgrounds [8]. Low socioeconomic groups also experience higher rates of food insecurity [8, 9]. Furthermore, low socioeconomic groups have poorer dietary intakes related to lower intakes of fruit and vegetables [9], less healthy food and beverages and similar intakes of discretionary foods compared to higher socioeconomic groups [10]. This is likely related, in part, to the unaffordability of recommended diets and the perception that healthy foods are more expensive than unhealthy foods [11, 12]. Consequently, there are higher rates of diet‐related disease amongst this population [9].

The affordability of healthy and equitable diets is a fundamental component of food security [10]. To improve food security and reduce diet‐related health inequities, continuing regular and comprehensive monitoring of food cost and affordability is essential to provide insights that can inform and guide policy initiatives. Within Australia, various ‘healthy food basket’ surveys have been utilised to assess food cost and affordability [13], however, differences between protocols and methods hindered comparability [13, 14]. Additionally, previous methods have not provided an accurate representation of the cost of ‘current’ Australian diets (i.e., the purchasing and eating behaviours of the population) compared to a ‘recommended’ diet that is aligned with the Australian Dietary Guidelines [14]. To address the urgent call for robust and standardised data, the Healthy Diets Australian Standardised Affordability and Pricing (HD‐ASAP) protocol was developed [14]. Aligning with the International Network for Food and Obesity/Non‐Communicable Diseases Research, Monitoring and Action Support (INFORMAS) ‘optimal approach’ [15], the five‐part protocol aims to compare the cost and affordability of both recommended diets (based on the Australian Dietary Guidelines) and current diets (based on reported intakes in the 2011–2013 Australian Health Survey National Nutrition and Physical Activity Survey [AHS NNPAS]) [14]. A modified version of the protocol, the low socioeconomic group HD‐ASAP (Low SEG HD‐ASAP) protocol [16], was developed to more closely align to the needs and personal experiences [17] of those in the lowest income quintile.

The Fowler electorate was selected for this investigation at the request of its Federal Member of Parliament, who expressed interest in obtaining information on the affordability of food to inform the enhancement of local‐level initiatives and support advocacy efforts at the federal level. Situated in South‐West Sydney, residents of the Fowler electorate experience particularly high rates of socioeconomic disadvantage [18]. Census data from 2021 revealed a majority of families comprised coupled families with children (48.6%) [19]. Single‐parent families (23.1%), particularly led by female single parents (81.5%), were also notably present, as were single‐person households (20.4%) [19]. As well as having high rates of individuals not in the labour force (49%), many residents live in rented accommodation (42.1%) and are culturally diverse (60.9% were born overseas, notably 31.8% of the population are from Vietnamese or Chinese backgrounds) [19]. It has been well documented that those at highest risk of food insecurity in Australia include unemployed people, single‐parent households, low‐income earners, rental households, as well First Nations, culturally and linguistically diverse (CALD) and socially‐isolated people [20]. Thus, given the social, cultural and economic dynamics of the Fowler electorate, investigation into food cost and affordability in the region is warranted.

Therefore, this study aimed to assess the cost, cost differential and affordability of recommended and current diets, including costs based on prices of both popular ‘standard brands’ and ‘cheapest alternative’ options for various household structures and income types within the Fowler electorate. The findings will generate actionable insights to inform policy initiatives to help improve the affordability and consumption of healthy foods and ultimately contribute to increasing the food security of residents in the Fowler electorate and, more broadly, low socioeconomic groups.

## 2 Methods

This cross‐sectional study applied the Low SEG HD‐ASAP protocol. The high rates of socioeconomic disadvantage across the Fowler electorate provided the rationale behind this choice. Modified from the original HD‐ASAP five‐part protocol, this protocol incorporates a current diet pricing tool based on reported dietary intakes of those in the lowest income quintile in Australia, food purchasing behaviours commonly used by low‐income households (i.e., shopping at budget supermarkets and purchasing the ‘cheapest alternative’ food options) and low‐minimum wage and welfare‐based household income sources [16]. The protocol modifications are further detailed elsewhere [16].

### 2.1 Household Composition

Three reference households from the HD‐ASAP protocols were selected to reflect the predominant household types in the Fowler electorate, including coupled families with children, female‐led single‐parent households and single‐person households [19]. These were labelled 1, 2 and 3, shown in Table 1.

**TABLE 1:** Reference households used in the study based on the Fowler demographics.

Reference household	Household composition
Household 1	Adult male (31–50 years), adult female (31–50 years), boy (14–18 years), child (4–8 years)
Household 2	Adult female (31–50 years), boy (14–18 years), child (4–8 years)
Household 3	Single unemployed person: adult male (31–50 years)

In the Low SEG HD‐ASAP adaptation of the original protocol, the definition of the reference children in Households 1 and 2 was expanded from a female (8 years) to a child (4–8 years) and from a male (14 years) to a male (14–18 years). This ensured that mean reported dietary intakes for the reference children, used to determine the current diet pricing tool, were sourced from a sufficient sample size in the 2011–2013 AHS NNPAS. The expanded age ranges are consistent with the age and gender groups identified in Australian Dietary Guidelines, as having the same nutritional requirements [16].

### 2.2 Standardised Diet Pricing Tools

The diet pricing tools applied contained the type and quantities of food and beverages for each reference household per fortnight (Table S1). The current diet pricing tool is based on the reported dietary intakes *of individuals in the lowest household income quintile* from the 2011 to 2013 Australian Health Survey National Nutrition and Physical Activity Survey [21]. The recommended diet pricing tool includes only the healthy foods of the current diet in larger quantities to align with the recommendation of the Australian Dietary Guidelines [22]. Foods and beverage items included in each diet are listed in Table 2.

**TABLE 2:** Food and beverage items included in the original healthy diets ASAP and modified low socioeconomic group healthy diets ASAP recommended and current diet‐pricing tools [17].

Recommended (healthy) diet	Current (unhealthy) diet
Water (bottled) Fruit: apples, bananas, oranges Vegetables: potatoes, broccoli, white cabbage, iceberg lettuce, onion, carrot, pumpkin, tomatoes, sweetcorn (canned), four bean mix (canned), diced tomatoes (canned), baked beans (canned), frozen mixed vegetables, frozen peas, salad vegetables in sandwich Grain (cereals): wholegrain cereal biscuits (Weet‐bix), rolled oats, cornflakes, wholemeal bread, white bread, white rice, white pasta, dry water crackers, bread in sandwich Lean meats and alternatives: beef mince and steak, lamb chops, cooked chicken, tuna (canned), eggs, peanuts (unsalted), meat in sandwich Milk, yoghurt and cheese: cheddar cheese (full fat, reduced fat), milk (full fat, reduced fat), yoghurt (full fat plain, reduced fat flavoured) Unsaturated oils and spreads: olive oil, sunflower oil, canola (margarine)	Healthy foods and beverages as per the seven food groups in the ‘Recommended Diet’ column; in reduced amounts reflecting reported intakes Discretionary (unhealthy) foods and beverages: – Beverages: sugar sweetened beverages, artificially sweetened beverages – Cereals, snacks and desserts: muffin, sweet biscuits, savoury crackers, confectionary, chocolate, potato crisps, muesli bar, mixed nuts (salted), ice cream, fruit salad (canned in juice) – Processed meats: beef sausages, ham – Spreads, sauces, condiments and ingredients: butter, tomato sauce, salad dressing, white sugar – Convenience meals: frozen lasagne, chicken soup (canned), frozen fish fillet (crumbed), instant noodles, meat and vegetable stew (canned) – Fast food: supreme pizza, plain beef pie, hamburger, potato chips/fries – Alcohol: beer (full strength), white wine (sparkling), red wine, whisky

### 2.3 Sample Location

Areas of the Fowler electorate were identified using Australian Bureau of Statistics map filtering of Statistical Area Level (SA2) and Commonwealth Electoral Divisions boundaries [23]. SA2 locations were stratified according to the Socioeconomic Indexes for Areas (SEIFA) Index of Relative Socioeconomic Disadvantage (IRSD) [18]. Of the 14 SA2 locations across Fowler, all but one (Quintile 3) were categorised as Quintile 1 (most disadvantaged). The Quintile 3 location extended across two electorates and was therefore excluded from the sampling frame. Five of the 13 Quintile 1 SA2 locations were randomly selected to include Liverpool East, Cabramatta‐Lansvale, Edensor Park, Greenfield Park‐Prairiewood and Fairfield East.

### 2.4 Data Collection Protocols

Food and beverage prices were collected by a trained student researcher in May 2024 using a paper‐based survey form and following the HD‐ASAP price collection protocols [14, 16] (Tables S2 and S3). Prices for ‘standard brands’ were collected from the following stores within 7 km of each SA2 location centre, identified using Google Maps: two large chain supermarkets, an independent grocer, a burger chain restaurant, a pizza restaurant, a liquor store, a local bakery and a fish and chip shop. The ‘cheapest alternatives’ prices were collected from the same two large supermarkets, liquor store and burger chain restaurant, however, a budget supermarket was surveyed in place of the independent grocer in each SA2 location. Additionally, the prices of three frozen food items (beef pie, hot chips and a supreme pizza) were collected from one of the large supermarkets as the cheapest alternative to the hot takeaway store items. This reflects the lower expenditure on takeaway foods within low socioeconomic groups [16]. Prices of unpackaged foods, including meats, fruits and vegetables, were the same value in both ‘standard brands’ and ‘cheapest alternatives’ price collections. Before data collection, permission was obtained from the national head offices of the large and budget supermarket chains. Verbal consent was obtained from store managers in each store at the time of each survey.

### 2.5 Calculation of Household Income

Low‐minimum and welfare‐dependent fortnightly household incomes were calculated for each household based on a set of assumptions, as per the low SEG HD‐ASAP protocol [16].

Low‐minimum income (low‐income) was calculated using minimum wage rates [24], tax payable [25] and any applicable welfare payments [26] (Table S4). Welfare‐dependent income was based on welfare payments only (Table S5).

### 2.6 Analysis and Reporting Methods

Food and beverage prices were entered into the HD‐ASAP online portal [27] and checked using the inbuilt validation system. Database algorithms generated Microsoft Excel spreadsheet results for each SA2 location. These were cross‐checked and then consolidated to capture the Fowler electorate as a whole. For each household, the mean fortnightly cost and standard deviation of recommended and current diets were calculated using both the ‘standard brands’ and ‘cheapest alternatives’ prices. The cost and proportion of the total spent on each food group, as well as the cost differentials between recommended and current diets, and between ‘standard brands’ and ‘cheapest alternatives’, were also calculated. Diet affordability was calculated as the proportion of household income required to purchase each diet specific to the household's size. Diets were considered unaffordable if they cost greater than 30% of household income [28] while those requiring 25%–30% of income indicated the household was at risk of ‘food stress’ [29].

## 3 Results

Food and beverage prices for recommended and current diets, including ‘standard brands’ and ‘cheapest alternatives’, were collected from five randomly selected SA2 locations across the Fowler electorate. In total, 43 stores were surveyed, occasionally crossing electorate boundaries if within seven kilometres of the SA2 locations centre. Due to the low number of stores within the vicinity of the SA2 locations, survey data from one independent grocer and one budget supermarket were utilised in two different SA2 locations. Furthermore, a charcoal chicken takeaway shop was substituted for a fish and chip shop for two of the SA2 locations, to collect the price of hot chips. This would accurately reflect the reality of the area, as residents would be more likely to visit these stores due to their proximity and convenience.

### 3.1 Cost of Recommended and Current Diets

**FIGURE 1:**
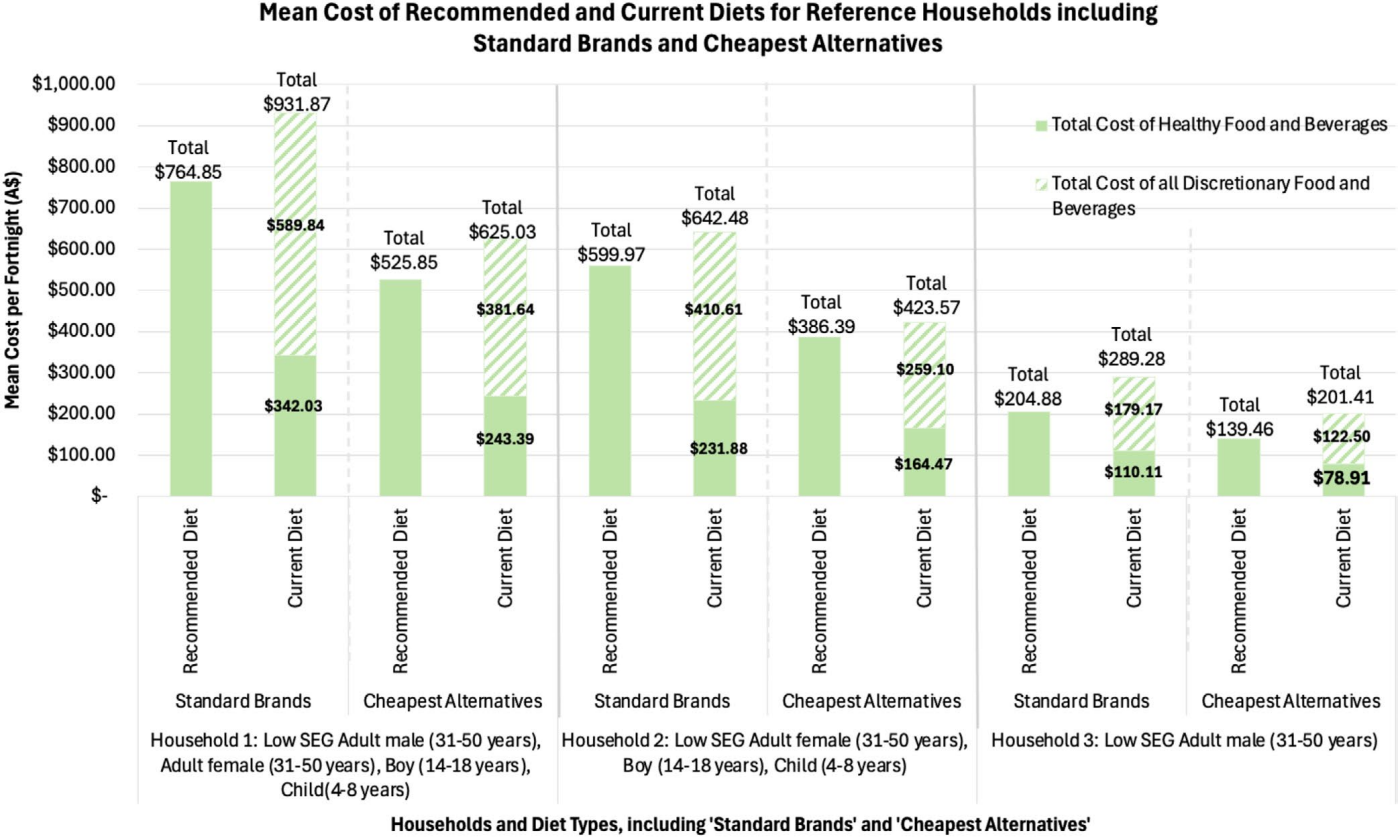
Mean fortnightly cost of recommended and current diets, for each household, including ‘standard brands’ and ‘cheapest alternatives’, across all Q1 locations (*n* = 5) within the Fowler Electorate, in May 2024.

The mean fortnightly cost of recommended and current diets for each household, including ‘standard brands’ and ‘cheapest alternative’ options, is presented in Figure 1. Detailed costs of food groups and components are provided in Table S6A–C.

Recommended diets were less expensive than current diets for all households. When pricing ‘standard brands’, recommended diets were less expensive by 18% ($167.02/fortnight) for Household 1, 13% ($82.51/fortnight) for Household 2 and 29% ($84.40/fortnight) for Household 3. When pricing ‘cheapest alternatives’, current diet costs reduced more than recommended diet costs due to the higher proportion of packaged food items in the current diet. This led to a decrease in the cost differential for Households 1 and 2 due to the greater reduction in current diet costs. However, recommended diets remained less expensive than current diets by 16% (equating to $99.18/fortnight) for Household 1, 9% (equating to $37.18/fortnight) for Household 2 and 31% (equating to $61.95/fortnight) for Household 3.

#### 3.1.1 Diet Costs When Pricing ‘Cheapest Alternative’ Options

Recommended diets were 31% less expensive for Households 1 and 2 ($239.00/fortnight and $173.58/fortnight, respectively), and 32% less expensive ($65.42/fortnight) for Household 3. Current diets were 33% less expensive ($306.84/fortnight) for Household 1, 34% less expensive ($218.91/fortnight) for Household 2 and 30% less expensive ($87.87/fortnight) for Household 3.

### 3.2 Affordability of Recommended and Current Diets

**FIGURE 2:**
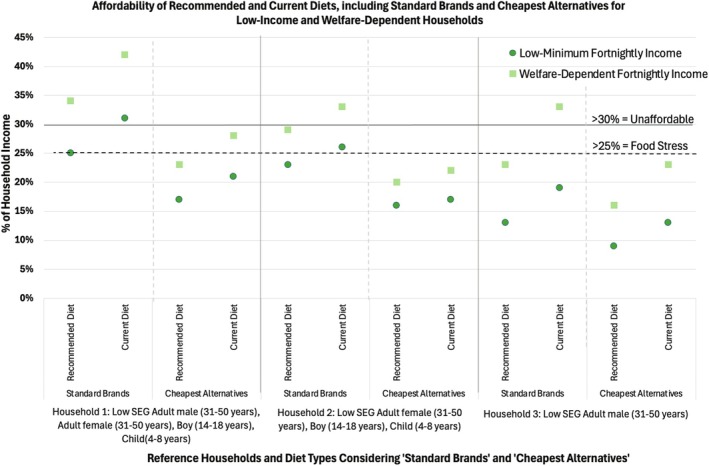
Affordability (% of household income) of recommended and current diets, including ‘standard brands’ and ‘cheapest alternatives’ for low‐income and welfare‐dependent households within the Fowler electorate, in May 2024.

The affordability (% of household income) of recommended and current diets, including ‘standard brand’ and ‘cheapest alternative’ options for low‐income and welfare‐dependent households, is displayed in Figure 2 and Table S6A–C.

#### 3.2.1 Diet Affordability for Low‐Income Households

When pricing ‘standard brands’ the affordability of the recommended diet fell into the ‘food stress’ range for Household 1 (25% of income), was close to the ‘food stress’ range for Household 2 (23% of income) and was in the affordable range for Household 3 (13% of income). The current diet was unaffordable for Household 1 (31% of income), fell into the ‘food stress’ range for Household 2 (26% of income) and was affordable for Household 3 (19% of income). When pricing ‘cheapest alternatives’, both recommended and current diets were affordable for all households, requiring between 9%–17% and 13%–21% of income, respectively.

#### 3.2.2 Diet Affordability for Welfare‐Dependent Households

When pricing ‘standard brands’ the recommended diet was unaffordable for Household 1 (34% of income), fell into the ‘food stress’ range for Household 2 (29% of income) and was affordable for Household 3 (23% of income). The current diet was unaffordable for all households, requiring 42% of income for Household 1 and 31% of income for both Households 2 and 3. When pricing ‘cheapest alternatives’, the recommended diet was affordable for all three households (20%–23% of income). The current diet fell into the ‘food stress’ range for Household 1 (28% of income) and was affordable for Households 2 and 3 (22% and 23% of income, respectively).

## 4 Discussion

The present study is the first to assess the cost, cost differential and affordability of recommended and current diets for various household structures and income types within the Fowler electorate, a socioeconomically disadvantaged region of NSW, Australia. Applying a standardised protocol, the present study found recommended diets were less expensive than current diets, but for welfare‐dependent and low‐income families with children, both diets were unaffordable or caused ‘food stress’ unless the ‘cheapest alternatives’ were bought.

These results could inform local, state and national policy initiatives aimed at improving the affordability and consumption of recommended diets, thereby enhancing the food security of residents in the Fowler electorate.

The finding that recommended diets were less expensive than current diets aligns with the results of previous applications of the various HD‐ASAP protocol frameworks [30]. This finding is likely partly attributable to the Goods and Services Tax (GST) exemption that Australia applies to basic healthy foods [31], and the inclusion of takeaway items and alcohol (particularly for adult males aged 31–50 years) within the current diet‐pricing tools [14, 16]. The present study identified that alcohol and takeaway items contributed to current diet costs by 7%–20% and 19%–27%, respectively. Furthermore, the inclusion of alcohol contributed to the greater cost differentials between recommended and current diets in Household 1 and, more conspicuously, Household 3, due to higher reported intakes by males compared to females. These findings confirm how the exclusion of these components could underestimate total diet costs, and indeed studies that have not considered these dietary components have suggested that ‘healthy diets’ are more expensive than ‘current diets’ [32, 33]. Furthermore, findings are comparable to non‐HD‐ASAP studies conducted within Australia [34, 35, 36, 37, 38, 39] and New Zealand [40]. Despite methodological differences, these studies demonstrated that ‘healthy’ diets were less expensive than ‘current’ or alternative diets [34, 35, 36, 37, 38, 39, 40].

Findings also revealed diet cost reductions for all households when the prices of packaged food and beverage items were sourced from the ‘cheapest alternative’ options. While recommended diets remained less expensive, the cost differential between recommended and current diets narrowed significantly in households with children, due to the greater cost reductions within current diets (33%–34%) in comparison to recommended diets (31%). This is likely a result of the widespread availability of unhealthier and ultra‐processed food and beverage items which are more prominently included in the current diet pricing tool being available to purchase at cheaper costs. Two methodically identical studies by Lewis et al. [16, 17] identified similar cost reduction results; however, also demonstrated circumstances where the cost differential between diets reversed, and current diets became less expensive than recommended diets—although only by AU$3–7/fortnight [16, 17]. The result of greater cost reductions when purchasing current diets is further supported by the findings of Chapman et al. [41], who assessed the cost differences between ‘branded’ and ‘generic branded’ food and beverage items (not diets). Results demonstrated greater average cost savings within discretionary items compared to other core recommended foods [41]. These collective results may contribute to the perception amongst low socioeconomic groups that healthy foods are more expensive than unhealthy foods [11, 12, 16].

In contrast, a HD‐ASAP study by Zorbas et al. [42] identified greater cost reductions for recommended diets (25%) compared to current diets (20%) when ‘generic branded equivalents’ were substituted. Notably, Zorbas et al. [42] applied the original HD‐ASAP protocol to reflect diet costs for the average population. Thus, variations in results are likely explained by the difference in current diet‐pricing tools (i.e., different quantities of discretionary items compared to healthy foods) and the different price collection methodology (i.e., exclusion of budget supermarkets and inclusion of hot takeaway items compared to frozen equivalents) [42]. Overall, while results highlight the financial benefits when purchasing ‘cheapest alternatives’, the promotion of these must be approached with caution as they may have unintentional repercussions, including cost savings being utilised to purchase more unhealthier food and beverages [16]. Furthermore, the impact on the food system regarding long‐term costs, consumer choice and market competition requires further consideration and investigation [43, 44].

Our study adds further evidence to the hypothesis that food costs and household income have a strong influence on diet affordability [45]. Our findings demonstrate that current diets were a greater financial burden on all households, particularly when purchasing ‘standard brands’.

Furthermore, despite recommended diets being less expensive, they remained unaffordable or caused ‘food stress’ for many welfare‐dependent and low‐income families with children unless the ‘cheapest alternatives’ were purchased. This aligns with a recent Australian systematic review [30] that identified that recommended diets were unaffordable for vulnerable population groups, including Aboriginal and Torres Strait Islander peoples, those on low or welfare‐dependent incomes and those residing in rural or remote areas. Studies by Lee et al. [16, 17] also demonstrated similar diet affordability estimates for low‐income and welfare‐dependent families with children; however, they further demonstrated that despite purchasing the ‘cheapest alternatives’, a recommended diet still caused ‘food stress’ for welfare‐dependent families of four [16, 17].

Our findings should be further considered in the context of the rising cost of living within Australia since 2020, where all basic living expenses, including food, housing and utilities, transport and healthcare costs, have increased [46]. While diet affordability is assessed against a benchmark of 30% [28], under financial constraints, the household food budget is perceived to be the most flexible expense and is likely to be reduced to compensate for other fixed expenses [9]. Within the Fowler electorate in 2021, almost one‐half of those renting accommodation experienced ‘rental stress’ (i.e., spending over 30% of household income on housing costs) [19]. Consequently, these households likely have less than 30% of income to spend on food. This exemplifies the economic challenges that may be experienced by many households within the Fowler electorate and highlights the increased risk of food insecurity.

Findings provide insights that can inform local‐level initiatives and programmes aimed at improving food security in the Fowler electorate. Several council‐supported low‐cost and free meal services and community pantries operate within the Fowler electorate [47]; however, there is little evidence of other local food access or supply initiatives, highlighting a gap that findings of this study can help inform.

In collaboration with key stakeholders, initiatives may encompass expanding community gardens, farmers markets and community pantries to improve vulnerable households' access to healthy food. Implementing culturally appropriate nutrition education programmes can improve residents' budgeting strategies [7] and understanding of how to grow, access and prepare affordable, healthy meals. Notably, the reduction of economic barriers would allow for greater success of these programmes [16]. While these strategies provide short‐term relief, they do not address the underlying social determinants of food insecurity. The broader implications of this study highlight the urgent need for stronger fiscal policy measures. Increasing household income through adequate welfare and income support payments would enable vulnerable populations to afford their basic needs, including nutritious food—an issue consistently advocated by the Australian Council of Social Service [48]. Studies have demonstrated the increased income supplements provided by the Australian Government during the COVID‐19 pandemic enabled low‐income and welfare‐dependent families to afford a healthy diet [45, 49]. A survey conducted during that period found that many welfare‐dependent individuals reported eating healthier foods as a result [50]. These examples highlight the positive effect of enhancing welfare and income support payments to adequate levels to ensure recommended diets are affordable. If recommended diets were affordable for all, this would lead to enhanced learning outcomes for children, improved workforce engagement and productivity, greater community participation, reduced social and diet‐related health inequalities and reduced strain on the healthcare system [16]. To improve food security amongst vulnerable population groups, ongoing monitoring of diet cost and affordability is essential to provide up‐to‐date evidence to inform policy and advocate for change.

The HD‐ASAP protocols have methodological limitations that have been described previously [14, 16]. While the diet‐pricing tools of the HD‐ASAP protocols aim to include food and beverages that are accessible, commonly consumed and culturally acceptable [14, 16], they may not be as representative of the eating and purchasing behaviours of households within locations with high rates of cultural diversity, such as the Fowler electorate. Future research should consider the adaptation of diet pricing tools to reflect culturally appropriate diets to better assess diet affordability within culturally diverse populations. Another methodological limitation is that the price collection protocols focus on larger supermarkets (where the majority of the diet can be purchased) and overlook smaller grocers, markets and independent stores, which may impact diet affordability estimates. Future studies within the Fowler electorate should consider including these stores to enable cost and affordability comparisons to further guide local‐level initiatives. Finally, this study focused on diet affordability and did not consider other components of food security, such as food accessibility. Considering the unintentional finding that identified a low number of supermarket stores across the Fowler electorate, future research should consider a mapping study of the local food environment to identify if access barriers to healthy foods exist. Such information could inform local and state government planning and policy initiatives aimed at improving food security within the Fowler electorate.

## 5 Conclusion

In a low socioeconomic region, despite recommended diets being less expensive than current diets, they were unaffordable or caused ‘food stress’ for many low‐income and welfare‐dependent families with children unless the ‘cheapest alternatives’ were purchased.

Implementation of local‐level initiatives and programmes to improve food security and ensure individuals have access to sufficient quality and quantities of healthy foods is required. Broader implications highlight the urgent need for greater fiscal policy efforts and ongoing monitoring of diet cost and affordability to improve food security and reduce diet‐related health inequities amongst vulnerable population groups.

## Author Contributions

Conceptualization: K.E.C., K.W., K.K. and M.L. Methodology: M.L. Formal analysis: S.D. Investigation: S.D. Resources: M.L. Data curation: S.D. and M.L. Writing – original draft preparation: S.D. Writing – review and editing: S.D., K.E.C., K.W., K.K. and M.L. Supervision: K.E.C., K.W., K.K. and M.L. Project administration: S.D. All authors have read and agreed to the published version of the manuscript.

## Ethics Statement

This study did not involve any human or animal subjects and therefore did not require ethical approval.

## Conflicts of Interest

The authors declare no conflicts of interest.

## Supporting information


**Data S1:** Supporting Information Tables.

## Data Availability

The data that support the findings of this study are available from the corresponding author upon reasonable request.
