# Supplementary material for: Where Cost of Food Hits Hardest: Investigation of Diet Cost and Affordability in a Low Socioeconomic Region of Australia
Source: Health Promot J Austr. 2025 Sep 2;36(4):e70092. doi: 10.1002/hpja.70092 (PMC12405604; doi:10.1002/hpja.70092)
Supplement: Supplementary file 1 — Data S1: Supporting Information Tables. [file HPJA-36-0-s001.pdf]

Supplementary Table 1: Food and beverage quantities per fortnight (not adjusted for edible/cooked portions) for Low SEG Healthy Diets ASAP recommended and current diet-pricing tools for each household rounded to the nearest mL or g.

	**Household 1** Low SEG Adult male (31-50 years), Adult female (31-50 years), Boy (14-18 years), Child (4-8 years)		**Household 2** Low SEG adult female (31-50 years), Boy (14-18 years), Child (4-8 years)		**Household 3** Low SEG Adult male (31-50 years)	
**Food Item**	**Current**	**Recommended**	**Current**	**Recommended**	**Current**	**Recommended**
**Energy (kJ/day)**	32,517 kJ	32,996 kJ	23,424 kJ	23,658 kJ	9093 kJ	9338 kJ
**Water (mL)**						
Water, bottled	3485	5296	2273	3275	1211	2021
**Fruit (g)**						
Apples	3638	5460	2808	4060	831	1400
Bananas	795	5460	614	4060	181	1400
Oranges	971	5460	560	4060	411	1400
Fruit salad, canned in juice	1544	0	1089	0	454	0
Total Fruit	9614	16380	7298	12180	2317	4200
**Vegetables and Legumes (g)**						
Potato, loose	1844	2320	955	1620	889	700
Broccoli, loose	389	1470	209	1120	180	350
White cabbage, loose	175	1470	137	1120	38	350
Iceberg lettuce, whole	704	1470	378	1120	326	350
Carrot, loose	618	2205	485	1680	134	525
Pumpkin	330	2205	289	1680	41	525
Onion, loose	106	1638	82	1218	24	420
Tomatoes, loose	654	1638	397	1218	257	420
Sweetcorn, canned	216	1160	179	810	37	350
Four bean mix, canned	61	1005	42	480	19	525
Diced tomatoes, canned	175	1638	110	1218	65	420
Baked Beans, canned	237	1005	96	480	141	525
Frozen mixed vegetables	746	1638	482	1218	264	420
Frozen peas	334	1638	128	1218	206	420
Total Vegetables and Legumes	7136	22500	4269	16200	2870	6353
**Grain (Cereal) Foods – Wholegrain and Refined (g)**						
Wholemeal bread, pre-packaged	870	4272	607	3152	263	1120
White bread, pre-packaged	3001	893	2177	669	824	224
Rolled oats	578	6648	559	4968	19	1680
Breakfast cereal, corn flakes	509	670	289	502	220	168
Breakfast cereal, wheat biscuits	243	2216	160	1656	83	560
White pasta	988	2042	580	1499	408	543

White rice	1904	2042	1248	1499	656	543
Dry wheat crackers, water crackers	89	781	70	585	19	196
Total Grain (Cereal) Foods	8336	19564	5837	14530	2499	5087
**Lean Meats and Poultry, Fish, Eggs, Nuts and Seeds (g)**						
Tuna, canned in oil	760	1841	553	1374	207	467
Beef mince, lean	163	1168	78	865	85	303
Lamb loin chops	333	1169	113	866	220	303
Beef rump steak	1042	1172	692	868	350	304
Eggs	884	2208	524	1648	360	560
Chicken, cooked whole	1093	1471	829	1098	264	373
Peanuts, roasted, unsalted	0	780	0	360	0	420
Total Lean Meats and Poultry, Fish, Eggs, Nuts and Seeds	4822	9809	3089	7079	1734	2783
**Milk, Yoghurt, Cheese and Alternatives**						
Cheddar cheese, full fat (g)	682	704	509	544	173	160
Cheddar cheese, reduced fat (g)	49	516	39	396	10	120
Milk, full fat (mL)	7301	6438	5328	4938	1973	1500
Milk, reduced fat (mL)	1839	12000	1301	9000	539	3000
Flavoured milk (mL)	2187	0	1375	0	812	0
Yoghurt, full fat, plain (g)	101	2576	69	1976	32	600
Yoghurt, flavoured reduced fat (g)	722	5100	597	3900	125	1200
Total Milk, Yoghurt, Cheese and Alternatives (g)	12881	27334	9218	20754	3663	6580
**Unsaturated Oils and Spreads (or foods from which these are derived)**						
Sunflower oil (mL)	15	291	7	160	7	131
Olive oil (mL)	15	291	7	160	7	131
Canola margarine (g)	197	412	149	226	48	186
Total Unsaturated Oils and Spreads (g)	227	994	163	546	63	448
**Discretionary Choices (g)**						
Chicken soup, canned	2219	0	876	0	1343	0
Muffin, commercial	922	0	666	0	256	0
Instant noodles, wheat based	227	0	196	0	31	0
White sugar	714	0	430	0	283	0
Cream-filled sweet biscuit, pre-packaged	628	0	441	0	187	0
Muesli bar, pre-packaged	339	0	315	0	23	0
Savoury flavoured	207	0	199	0	8	0

crackers						
Nuts, mixed, salted	262	0	197	0	65	0
Confectionary	396	0	365	0	30	0
Chocolate	359	0	280	0	79	0
Potato crisps, pre-packaged	650	0	608	0	42	0
Salad dressing	211	0	161	0	50	0
Tomato sauce	511	0	375	0	136	0
Beef sausages	1036	0	670	0	365	0
Butter	195	0	109	0	85	0
Ham	143	0	110	0	33	0
Frozen lasagne, pre-packaged	3684	0	2828	0	856	0
Fish fillet crumbed, pre-packaged	433	0	247	0	186	0
Ice cream	1307	0	1077	0	230	0
Total Discretionary	17109	0	12377	0	4728	0
**Alcoholic Beverages (mL)**						
Beer, full strength	5060	0	545	0	4515	0
White wine, sparkling	546	0	433	0	114	0
Whisky	453	0	371	0	82	0
Red wine	519	0	218	0	301	0
Total Alcoholic Beverages	6578	0	1567	0	5012	0
**Takeaway foods (g)**						
Pizza, commercial	1800	0	1546	0	254	0
Plain beef pie, commercial	1554	0	1099	0	456	0
Hamburger, commercial	2710	0	1896	0	814	0
Potato chips, commercial	833	0	631	0	202	0
Total Takeaway foods	6897	0	5172	0	1726	0
**Sugar sweetened beverages (mL)**						
Sugar-sweetened soft drink	16288	0	13598	0	2690	0
**Artificially sweetened beverages**						
Artificially sweetened soft drink	1406	0	561	0	845	0
**Items allocated to more than one food group**						
Sandwich, pre-made, white bread, chicken and salad (g)	462	360	442	360	20	160
Canned meat and vegetable casserole (g)	786	0	305	0	481	0
Orange fruit juice (mL)	5331	0	4453	0	879	0

Supplementary Table 2: The Healthy Diets ASAP food price data collection protocol.

1. Record the usual price of an item, i.e. do not collect the sale/special price unless it is the only price available (if so, note in the comment column)
2. Look for the specified brand and specified size for each food item, and record the price
   • If the specified brand is not available: Choose the cheapest brand (non-generic) available in the specified size. Note this brand in the "Your brand" column.
   • If the specified size is not available: Choose the nearest larger size in the specified brand. If a larger size is not available, choose the nearest smaller size. Note this size in the "Your size" column.
   • If both the specified brand and specified size are not available: Choose the cheapest in the nearest larger size of another brand (non-generic). If a larger size is not available, choose the nearest smaller size.
   • If multiple brands are specified, record the price of the cheapest one and note the brand in the "Your brand" column
   • If the item is only available in a generic form (e.g. Home Brand, Coles, Woolworths Select, Black and Gold) choose the most expensive generic item in the specified size. If the specified size is not available, choose the nearest larger size. If a larger size is not available, choose the nearest smaller size. Note the generic name in the "Your brand" and the size in the "Your size" columns.
3. Loose produce: choose the usual cheapest price per kg of the variety not on special. If the only variety available is on special, record the special price and note in the comment's column.
4. Peanuts: choose the branded packet size closest to 250 g. If packaged, roasted, unsalted peanuts are not available, record the price of the loose 'bulk scoop & weigh' roasted, unsalted peanuts per 100g.
5. Check all data are collected and recorded as above, before leaving store.

Supplementary Table 3: The Low SEG Healthy Diets ASAP food price data collection protocol.

1. When collecting ‘cheapest option’ prices, select the cheapest equivalent product from all brands including ‘own brands’ in the specified size.
2. For items including pizza, plain beef pie, and hot chips, usually sourced from other stores, the price of a frozen equivalent item from the supermarket is collected, selecting the cheapest option from all brands, including ‘own brands,’ in the specified size.
3. The takeaway burger should be priced from the burger restaurants as per the original protocol.
4. If the specified size is not available, choose the nearest larger size.
5. If a larger size is not available, choose the nearest smaller size.

Supplementary Table 4: Low-minimum income calculations and assumptions for each reference household in April 2024.

	**Household 1: Two parents with two children** Adult male, adult female, 14yr boy, 8yr girl		**Household 2: Single parent with 2 children** Adult female, 14yr boy, 8yr girl		**Household 3: Single unemployed person** Adult male	
**Assumptions**	• The adult male works on a permanent basis at national minimum wage for 38 hours a week ($23.23/hr)<br>• The adult female works on a part-time basis at national minimum wage ($23.23/hr) for 6 hours a week<br>• Both children attend school and are fully immunised<br>• None of the family are disabled<br>• The family has some emergency savings that earn negligible interest<br>• The family is privately renting a 3 bedroom house at $450/week	**Amounts per fortnight Apr 2024**	• The adult female works on a casual basis at national minimum wage ($26.71/hr ($23.23/hr + 15% casual loading)) for 25 hours a week for 39 weeks per year (not during school holidays)<br>• The adult female does not receive child support from the children's father<br>• Both children attend school and are fully immunised<br>• None of the family are disabled<br>• The family does not have savings or investments<br>• The family is privately renting their home at $450/week	**Amounts per fortnight Apr 2024**	• Works on a permanent basis at national minimum wage for 38 hours a week ($23.23/hr)<br>• Is not studying/training<br>• Is not disabled<br>• Has no dependent children<br>• Does not have savings or investments<br>• Is renting a room in 3-bedroom house at $150/week ($450/3)	**Amounts per fortnight Apr 2024**
**INCOME (fortnightly)**						
Paid employment- adult male	$23.23/hr for 38h/week	$1765.48	N/A	-	$23.23/hr for 38h/week	$1,765.48
Paid employment- adult female	$23.23/hr for 6h/week	$278.76	$26.71/h/25h per week/39 weeks	$1,001.63	N/A	-
JobSeeker Allowance	N/A	-	N/A	-	N/A	-
Parenting Payment	$387.40/fortnight	$387.40	$685.14/fortnight	$685.14	N/A	-
Family Tax Benefit A fortnightly payment	$490.84/fortnight	$490.84	$490.84/fortnight	$490.84	N/A	-
Family Tax Benefit A annual supplement	$879.65/child/year	$67.67	$879.65/child/year	$67.67	N/A	-
Family Tax Benefit B fortnightly payment	$43.54/fortnight	$43.54	$126.56/fortnight	$126.56	N/A	-
Family Tax Benefit B annual supplement	$430.70/year/family	$16.57	$430.70/year/family	$16.57	N/A	-
Age Pension fortnightly payment	N/A	-	N/A	-	N/A	-
Age Pension Fortnightly Supplement	N/A	-	N/A	-	N/A	-
Total Clean Energy Supplement (from all payments)	Included in FTB and jobseeker estimator	-	Included in FTB and jobseeker estimator	-	N/A	-
Rent Assistance	$221.20/fortnight	$221.20	$221.20/fortnight	$221.20	N/A	-
**INCOME TAX PAID**	Tax + Medicare levy, less low-income tax offset	$230.45	Tax + Medicare levy, less low-income tax offset	$172.79	Tax + Medicare levy, less low-income tax offset	$230.45
**FORTNIGHTLY INCOME TOTAL**		**$3,041.00**		**$2436.81**		**$1,535.03**

Supplementary Table 5: Welfare-dependent income calculations and assumptions for each reference household in April 2024

	**Household 1: Two parents with two children**<br>Adult male, adult female, 14yr boy, 8yr girl		**Household 2: Single parent with 2 children**<br>Adult female, 14yr boy, 8yr girl		**Household 3: Single unemployed person**<br>Adult male	
**Assumptions**	• The adult male is unemployed<br>• The adult female is unemployed<br>• Both children attend school and are fully immunised<br>• None of the family are disabled<br>• The family has some emergency savings that earn negligible interest<br>• The family is privately renting a 3-bedroom house at $450/week	**Amounts per fortnight Apr 2024**	• The adult female is unemployed and looking for work<br>• The adult female does not receive child support from the children's father<br>• Both children attend school and are fully immunised<br>• None of the family are disabled<br>• The family does not have savings or investments<br>• The family is privately renting their home at $450/week	**Amounts per fortnight Apr 2024**	• Has no paid employment but is looking for work<br>• Is not studying/training<br>• Is not disabled<br>• Has no dependent children<br>• Does not have savings or investments<br>• Is renting a room in 3-bedroom house at $150/week ($450/3)	**Amounts per fortnight Apr 2024**
**INCOME (fortnightly)**						
Paid employment- adult male	N/A	-	N/A	-	Nil	-
Paid employment- adult female	N/A	-	Nil	-	N/A	-
JobSeeker Allowance	$706.20/fortnight	$706.20	N/A	-	$762.70/fortnight	$762.70
Parenting Payment	$706.20/fortnight	$706.20	$999.70/fortnight	$999.70	N/A	-
Family Tax Benefit A fortnightly payment	$490.84/fortnight	$490.84	$490.84/fortnight	$490.84	N/A	-
Family Tax Benefit A annual supplement	$879.65/child/year	$67.67	$879.65/child/year	$67.67	N/A	-
Family Tax Benefit B fortnightly payment	$35.56/fortnight	$35.56	$126.56/fortnight	$126.56	N/A	-
Family Tax Benefit B annual supplement	$430.70/year/family	$16.57	$430.70/year/family	$16.57	N/A	-
Age Pension fortnightly payment	N/A	-	N/A	-	N/A	-
Age Pension Fortnightly Supplement	N/A	-	N/A	-	N/A	-
Total Clean Energy Supplement (from all payments)	Included in FTB and jobseeker estimator	-	Included in FTB and jobseeker estimator	-	$8.80	$8.80
Rent Assistance	$221.20/fortnight	$221.20	$221.20/fortnight	$221.20	$115.50/fortnight	$115.50
INCOME TAX PAID	Nil		Nil		Nil	
**FORTNIGHTLY INCOME TOTAL**		**$2,244.23**		**$1,922.53**		**$887.00**

Supplementary Table 6A: Mean fortnightly diet cost and affordability by food group and food group components of recommended and current diets, for Low SEG adult male (31-50 years), adult female (31-50 years), boy (14-18 years), child (4-8 years) (Household 1), across all quintile 1 locations (n=5) within the Fowler electorate in May 2024.

	**Low SEG Standard Brands**				**Low SEG Cheapest Alternatives**			
	**Recommended**		**Current**		**Recommended**		**Current**	
**Food group and food group component**	**Mean cost (A$) ± SD**	**Proportion of total diet cost (%)**	**Mean cost (A$) ± SD**	**Proportion of total diet cost (%)**	**Mean cost (A$) ± SD**	**Proportion of total diet cost (%)**	**Mean cost (A$) ± SD**	**Proportion of total diet cost (%)**
Water, bottled	21.68 ± 2.77	2.84	14.27 ± 1.82	1.53	9.15 ± 2.10	1.74	6.02 ± 1.38	0.96
Fruit	80.67 ± 13.57	10.55	48.90 ± 4.48	5.25	74.55 ± 7.43	14.18	39.07 ± 2.56	6.25
Vegetables (& legumes)	140.17 ± 21.71	18.33	52.39 ± 5.60	5.62	107.64 ± 7.02	20.47	42.53 ± 2.32	6.80
Grain (cereal) foods	136.71 ± 8.68	17.87	52.07 ± 5.55	5.59	61.74 ± 0.76	11.74	28.72 ± 0.49	4.60
Lean meats, poultry, fish, eggs, nuts, seeds & alternatives	231.49 ± 8.50	30.27	108.63 ± 3.07	11.66	180.11 ± 6.76	34.25	84.81 ± 4.90	13.57
Milk, yoghurt, cheese & alternatives	139.99 ± 4.34	18.30	63.25 ± 1.99	6.79	84.95 ± 3.60	16.15	40.89 ± 4.79	6.54
Unsaturated oils & spreads	14.14 ± 1.20	1.85	2.52 ± 0.13	0.27	7.71 ± 0.62	1.47	1.35 ± 0.03	0.22
Artificially sweetened beverages			4.44 ± 0.41	0.48			1.33 ± 0.03	0.21
Sugar sweetened beverages			51.38 ± 4.71	5.51			16.63 ± 4.40	2.66
Takeaway foods			216.01 ± 6.88	23.18			158.92 ± 0.95	25.43
Alcoholic beverages			101.05 ± 1.97	10.84			70.88 ± 4.28	11.34
All other discretionary choices			216.96 ± 14.69	23.28			133.87 ± 10.71	21.42
**Total Mean Diet Cost (A$)** ± SD	**764.85** ± 46.51	100	**931.87** ± 38.48	100	**525.85** ± 20.47	100	**625.03** ± 19.95	100
Fruit, vegetables & legumes	220.84 ± 28.87	28.87	101.29 ± 8.86	10.87	182.19 ± 11.73	34.65	81.60 ± 3.85	13.06
All healthy food and beverages	764.85 ± 46.51	100	342.03 ± 19.17	36.70	525.85 ± 20.47	100	243.39 ± 10.44	38.94
All discretionary food and beverages			589.84 ± 20.67	63.30			381.64 ± 11.26	61.06
**Diet affordability (% of fortnightly household income)**								
**Income category and amount (A$)**								
Low-minimum household income ($3041.00)	25%		31%		17%		21%	
Welfare dependent household income ($2,244.23)	34%		42%		23%		28%	

Supplementary Table 6B: Mean diet cost and affordability by food group and food group components of recommended and current diets per fortnight, for Low SEG adult female (31-50 years), boy (14-18 years), child (4-8 years) (Household 2), across all quintile 1 locations (n=5) within the Fowler electorate, in May 2024.

Food group and food group component	**Low SEG Standard Brands**				**Low SEG Cheapest Alternatives**			
	**Recommended**		**Current**		**Recommended**		**Current**	
	**Mean cost (A$) ± SD**	**Proportion of total diet cost (%)**	**Mean cost (A$) ± SD**	**Proportion of total diet cost (%)**	**Mean cost (A$) ± SD**	**Proportion of total diet cost (%)**	**Mean cost (A$) ± SD**	**Proportion of total diet cost (%)**
Water, bottled	13.41 ± 1.71	2.39	9.31 ± 1.19	1.45	5.66 ± 1.30	1.46	3.93 ± 0.90	0.93
Fruit	59.98 ± 10.09	10.71	36.33 ± 3.23	5.65	55.43 ± 5.52	14.35	29.11 ±1.87	6.87
Vegetables (& legumes)	100.78 ± 16.45	18.00	32.13 ± 3.05	5.00	78.64 ± 5.25	20.35	26.87 ± 1.41	6.34
Grain (cereal) foods	101.55 ± 6.45	18.13	38.70 ± 3.85	6.02	45.70 ± 0.56	11.83	21.91 ± 0.34	5.17
Lean meats, poultry, fish, eggs, nuts, seeds & alternatives	169.56 ± 5.63	30.28	68.10 ± 2.10	10.60	131.85 ± 4.93	34.12	52.26 ± 3.05	12.34
Milk, yoghurt, cheese & alternatives	106.92 ± 3.31	19.09	45.55 ± 1.42	7.09	64.88 ± 2.78	16.79	29.45 ± 3.22	6.95
Unsaturated oils & spreads	7.77 ± 0.66	1.39	1.76 ± 0.08	0.27	4.23 ± 0.34	1.10	0.94 ± 0.02	0.22
Artificially sweetened beverages			1.77 ± 0.16	0.28			0.53 ± 0.01	0.13
Sugar sweetened beverages			42.90 ± 3.93	6.68			13.88 ± 3.67	3.28
Takeaway foods			161.57 ± 5.11	25.15			115.58 ± 0.67	27.29
Alcoholic beverages			42.71 ± 1.61	6.65			31.25 ± 1.72	7.38
All other discretionary choices			161.67 ± 11.03	25.16			97.86 ± 6.91	23.10
**Total Mean Diet Cost (A$)** ± SD	**559.97** ± 34.08	100	**642.48** ± 27.13	100	**386.39** ± 15.06	100	**423.57** ± 13.04	100
Fruit, vegetables & legumes	160.76 ± 21.68	28.71	68.46 ± 5.52	10.65	134.07 ± 8.76	34.70	55.98 ± 2.63	13.22
All healthy food and beverages	559.97 ± 34.08	100	231.88 ± 12.62	36.09	386.39 ± 15.06	100	164.47 ± 6.95	38.83
All discretionary food and beverages			410.61 ± 15.62	63.91			259.10 ± 7.34	61.17
**Diet affordability (% of fortnightly household income)**								
**Income category and amount (A$)**								
Low-minimum household income ($2436.81)	23%		26%		16%		17%	
Welfare-dependent household income ($1,922.53)	29%		33%		20%		22%	

Supplementary Table 6C: Mean diet cost and affordability by food group and food group components of recommended and current diets per fortnight, for Low SEG adult male (31-50 years) (Household 3), across all quintile 1 locations (n=5) within the Fowler electorate, in May 2024.

	Low SEG Standard Brands				Low SEG Cheapest Alternatives			
	Recommended		Current		Recommended		Current	
**Food group and food group component**	**Mean cost (A$) ± SD**	**Proportion of total diet cost (%)**	**Mean cost (A$) ± SD**	**Proportion of total diet cost (%)**	**Mean cost (A$) ± SD**	**Proportion of total diet cost (%)**	**Mean cost (A$) ± SD**	**Proportion of total diet cost (%)**
Water, bottled	8.27 ± 1.06	4.04	4.96 ± 0.63	1.71	3.49 ± 0.80	2.50	2.09 ± 0.48	1.04
Fruit	20.68 ± 3.48	10.10	12.56 ± 1.26	4.34	19.11 ± 1.90	13.71	9.97 ± 0.71	4.95
Vegetables (& legumes)	39.39 ± 5.27	19.23	20.27 ± 2.56	7.01	29.00 ± 1.77	20.80	15.66 ± 0.91	7.77
Grain (cereal) foods	35.16 ± 2.23	17.16	13.37 ± 1.71	4.62	16.05 ± 0.20	11.51	6.81 ± 0.16	3.38
Lean meats, poultry, fish, eggs, nuts, seeds & alternatives	61.93 ± 2.96	30.23	40.53 ± 1.11	14.01	48.27 ± 1.83	34.61	32.55 ± 1.86	16.16
Milk, yoghurt, cheese & alternatives	33.07 ± 1.02	16.14	17.70 ± 0.58	6.12	20.07 ± 0.83	14.39	11.44 ± 1.60	5.68
Unsaturated oils & spreads	6.37 ± 0.54	3.11	0.73 ± 0.04	0.25	3.47 ± 0.28	2.49	0.39 ± 0.02	0.19
Artificially sweetened beverages			2.67 ± 0.24	0.92			0.80 ± 0.02	0.40
Sugar sweetened beverages			8.49 ± 0.78	2.93			2.75 ± 0.73	1.36
Takeaway foods			54.46 ± 1.80	18.83			43.36 ± 0.29	21.53
Alcoholic beverages			58.36 ± 0.36	20.17			39.64 ± 2.68	19.68
All other discretionary choices			55.19 ± 3.68	19.08			35.95 ± 3.88	17.85
**Total Diet Cost (A$)** ± SD	**204.88** ± 12.52	100	**289.28** ± 11.36	100	**139.46** ± 5.42	100	**201.41** ± 7.30	100
Fruit, vegetables & legumes	60.07 ± 7.20	29.32	32.83 ± 3.37	11.35	48.12 ± 2.97	34.50	25.63 ± 1.24	12.72
All healthy food and beverages	204.88 ± 12.52	100	110.11 ± 6.61	38.06	139.46 ± 5.42	100	78.91 ± 3.50	39.18
All discretionary food and beverages			179.17 ± 5.07	61.94			122.50 ± 4.59	60.82
**Diet affordability (% of fortnightly household income)**								
**Income category and amount (A$)**								
Low-minimum household income ($1,535.03)	13%		19%		9%		13%	
Welfare-dependent household income ($887.00)	23%		33%		16%		23%	
